# Bibliometric analysis of horticultural crop secondary metabolism

**DOI:** 10.1016/j.heliyon.2024.e26079

**Published:** 2024-02-14

**Authors:** Şenol Çelik

**Affiliations:** Biometry Genetics Unit, Department of Animal Science, Agricultural Faculty, Bingöl University, Bingöl, Turkey

**Keywords:** Bibliometry, Horticultural crop, Citation, Publish

## Abstract

The goal of the study was to examine the trends in recent years by analyzing 750 studies, 3619 authors, and 166 sources with the statement "Horticultural Crop Secondary Metabolism" in the article title published within the scope of SCI-Expanded and “Scopus” journals in between the years 2010 and 2023. In this case, the Web of Science Core Collection database was scanned under the heading "Horticultural Crop Secondary Metabolism", and bibliometric information was gathered. In order to advance research on horticulture crops, current problems and recommend solutions within "Horticultural Crop Secondary Metabolism" were identified in this study. The number of publications, publication kinds, reference analyses, total citations per year, most common words, most often cited local authors, most pertinent affiliations, and most pertinent sources were all examined in relation to the research. According to the findings, Horticulture Research, Frontiers in Plant Science, Plant Physiology and Biochemistry: PPB, Scientific Reports, and BMC Genomics are the journals that publish the most papers on "Horticultural Crop Secondary Metabolism". The phrases "gene expression regulation plant", "transcriptome", and "plant proteins" are used most frequently. Because of this, the increase of bibliometrics study can be very beneficial by serving as a catalyst for horticulture crop research.

## 1 Introduction

Abiotic stress conditions (drought, salinity, heavy metals, and temperature extremities) have a significant impact on horticultural crops. This is regarded as one of the most serious dangers to human health [1]. Plant growth, metabolism, and crop output have all been demonstrated to be drastically altered by abiotic stressors and environmental changes. Plants produce a wide range of (plant) secondary metabolites (PSM) from primary metabolites, frequently in response to abiotic environmental cues [[2], [3], [4]]. Salicylic acid, according to Ref. [1], is an essential bio-stimulator involved in the regulation of the growth and developmental phases of horticultural crops. The usage of even tiny doses of salicylic acid can increase the production of horticultural crops.

According to Asati and Yadav [5], there is significant diversity among regional horticultural species, including variance in plant type, morphological and physiological properties, reactivity to diseases and pests, adaptation, and distribution in the India's Northeast. Arunachal Pradesh, Assam, Manipur, Meghalaya, Mizoram, Nagaland, Tripura, and Sikkim make up India's North Eastern region. Aside from nutritional benefits, several regional horticulture products are employed in rural regions for medical purposes, money generation, and poverty alleviation programs.

Secondary metabolism should be investigated in important products such as tomatoes, potatoes, apples and Chili peppers in terms of human consumption, food need and economic value. Tomatoes are one of the most widely grown crop plants on the planet, with fields and greenhouses all over the world. Breeding using recognized features of wild species can improve farmed crop stress tolerance [6]. The effect of grafting has also been extensively investigated in the tomato, one of the world's most significant horticultural crops. In general, these effects include reducing the effects of soil-borne illnesses, boosting plant vigor and crop yield under normal growth circumstances, and inducing tolerance to abiotic stresses [[7], [8], [9]]. *Lycopersicon esculentum* is an important horticultural crop grown all over the world that has numerous health benefits [10].

The fourth-most important crop in the world for both human consumption and commercial food processing is the potato (*Solanum tuberosum* L). The potato has grown in popularity due to its high nutritional content and ease of multiplication through vegetative reproduction. However, farmed potatoes, like many other plants, are subjected to a variety of abiotic and biotic stressors, particularly as a host for a wide range of diseases [11]. One of the most challenging issues influencing potato production is that potatoes are a vegetatively propagated crop, meaning that they are prone to virus infection during propagation [12]. Anthocyanin and proanthocyanidin are essential components of plant secondary metabolism. Despite the identification of several transcription factors involved in anthocyanin and proanthocyanidin production in earlier research, the effects of MADS-box transcription factors in apple remain unknown [13].

An integral part of China's fruit tree industry is the apple, one of the most important fruit income crops in temperate regions. Apple trees with red flesh have twigs and young leaves that are reddish-purplish red, vibrant purplish-red blooms, and maturing fruits that maintain their purplish-red color. These trees are considered striking and unique due to the colors of their fruits, flowers, and foliage. The hue of the red-fleshed apple fruit is directly correlated with its anthocyanin content. Flavonoids called anthocyanins are crucial for growth and development as well as secondary metabolism [14].

The Food and Agriculture Organization of the United Nations (FAO) lists the chili pepper (*Capsicum* spp.) as one of the 50 primary food items and one of the most commercially significant horticulture crops in the world. Globally, there has been an increase in the use of chili fruits as a condiment or as a fresh meal [15]. The crop's secondary metabolism yields nutraceutical qualities that contribute to its commercial significance [16].

Secondary metabolism is important in maintaining all of the systems of plants functioning properly and in making plants resistant to abiotic and biotic stress. They are also utilized to inhibit feeding, as toxins, or as precursors to physical defense systems [17]. Secondary metabolism is essential for the normal operation of all plant systems, as well as for defense mechanisms. They defend themselves against herbivores, pests, and infections. Secondary metabolites are also utilized to inhibit consumption, as toxins, or as precursors to physical defense systems. Secondary metabolites include alkaloids, cyanogenic glycosides, flavonoids, terpenoids, and phenolic chemicals [18]. Secondary metabolites are also employed as dyes, glues, fibers, oils, waxes, flavoring agents, medicines, and perfumes, and they are thought to be a source of new natural medications, antibiotics, insecticides, and herbicides [19].

Research has been done on how the transcriptome and primary and secondary metabolites in the leaves of both cultivated and wild tomatoes respond to different abiotic stresses, such as nitrogen shortage, freezing or warmer temperatures, intense light, and combinations of these. In both species, nitrogen shortage caused the strongest reactions and, in particular, boosted secondary metabolism, however in the cultivated tomato to a far greater extent [6]. Terpenoids, flavonoids, and anthocyanins are examples of secondary metabolism in plants that occurs as a result of the plant defense mechanism to fend against viral invasion [20]. Therefore, a multitude of possible resistance resources for the creation of resistant cultivars are provided by plant basal resistance genes. Secondary metabolites in plants have a variety of tasks, including disease, insect, and herbivore defense, stress response, and modulating organismal relationships.

Bibliometric methodologies may be used to assess the intellectual structure, knowledge regions, particular research subjects and methods, and maturity levels of issues in a certain discipline or publication. Bibliometrics is a set of statistical methods for evaluating the publication performances of authors, institutions, and countries using data from written sources such as journals, books, and articles (e.g., citations, authors, keywords, statistical methods, and so on) and for investigating the evolution of disciplines by mapping their structures and dynamics on databases [21]. Bibliometric analysis is the statistical examination of scientific research papers and is an effective approach for assessing how an area of study begins and evolves [22].

Bibliometric analysis gained popularity after eminent English scientist Allen Richard coined the word "bibliometrics" instead of "statistical bibliography" in 1969 [23]. Bibliometric approaches may be used to statistically predict the character and direction of scientific communication in scientific publications based on citations of other sources and the bibliography of the work. Bibliometric analysis has been described as "the application of statistical and mathematical methods to books and other communication tools" by Pritchard [24]. Bibliometric analysis allows for the discovery of the present conditions by analyzing data from publications [25].

Bibliometrics is the quantitative examination of certain features of publications, such as the author, subject, publishing information, referenced sources, and so on. In bibliometric analysis, quantitative and statistical approaches are employed to characterize publishing trends in a certain field or in the literature [26].

According to Han et al. [27], bibliometric analysis is a computer-based approach for examining the current literature on a certain topic and establishing networks among this research. The Author Footprint Index (AFI) is a computation approach used to offer information about the frequency with which researchers see and browse articles [28].

The purpose of this study was to identify the most common study areas of horticulture crop secondary metabolism, the most common time periods, and the most essential publications of the selected time period. By conducting this analysis, it is aimed to understand the issue systematically and identify solution suggestions. By illuminating the research related to multimodal horticultural crop usage, this study is anticipated to provide a valuable contribution to the body of literature.

## 2 Methodology

### 2.1 Data collection

The studies in this article were assessed using the bibliometric analysis method. The Web of Science core collection database and publications included in the Science Citation Index-Expanded (SCI-EXPANDED), Social Sciences Citation Index (SSCI), Arts and Humanities Citation Index (AHCI), and Emerging Sources Citation Index (ESCI) indexes provided the study's data. All source publication searches and data download were performed on December 22nd, 2023. The search formula "horticultural OR crop" AND "secondary metabolism" was used. It was limited the publication time range from January 1st, 2010 to December 22nd, 2023, the document types to articles or reviews and the document language to English. The search yielded a total of 750 papers. It was extracted some important information of these articles such as title, keywords, authors, institutions, countries, publication year, publishing journals and saved them in plain files for further analysis. The bibliometric study was conducted using the R software's Bibliometrix package [29].

### 2.2 Methods

The link between the number of contributing writers in an article and the frequency with which their respective names are referenced is examined when AFI is computed. As a computation approach, the formula below is used.

$$AFI=1-\frac{Total\;number\;of\;authors\;in\;the\;field\;}{Total\;number\;of\;co-authors\;in\;the\;field}$$

Another index value based on author collaboration is the Collaboration Index (CI). This index is computed using numerous authors' papers in the subject [30].

$$CI=\frac{Authors\;of\;multi\;authored\;documents\;}{Multi\;authored\;documents}$$

Collaboration networks show collaborations between persons or organizations, such as nations, authors, or journals, and a social network. When looking at partner-ships for countries, a country x country adjacency matrix determined for co-publication frequency was employed. The network analysis based on co-publishing is formed as follows, based on the modularity score Q [31,32].

$$Q=\frac{1}{2h}\sum_{ii^{\prime }}\left[a_{ii^{\prime }}-\frac{\delta_{i}\delta_{i^{\prime }}}{2h}\right]s_{i}s_{i^{\prime }}$$

Here, $\delta_{i}$ indicates the degree of node i' in the formula, h represents the total number of edges, and $s_{i}$ represents the membership node to a community [33].

## 3 Results

According to the data from the WOS database, a total of 750 papers on horticulture crop secondary metabolism were published, 81 of which were articles and four of which were comparative studies. 3619 authors in all contributed to the study (Table 1).

**Table 1:** Main information about data.

Description	Results
Timespan	2010:2023
Sources (Journals, Books)	166
Documents	750
Annual Growth Rate %	22.08
Document Average Age	4.71
Keywords Plus (ID)	1081
Author's Keywords (DE)	2036
Authors	3619
Authors Collaboration	
Single-authored docs	4
Co-Authors per Doc	7.64
Comparative Study	29
Journal Article	718
Evaluation study	2
Review	1

### 3.1 Publication information

**Fig. 1:**
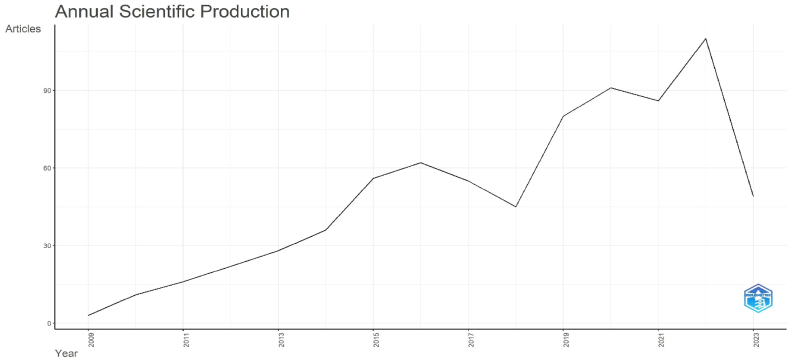
Annual scientific production.

There were 750 entries identified in the Web of Science database on December 22, 2023 while searching with the term "horticultural OR crop" AND "secondary metabolism". 2010 to 2023 were covered by this dataset (Table 2 and Fig. 1). Over that time span, there were annual peaks and valleys in the number of publications. In 2022, there were more papers published than in 2019. With 110 completed studies, 2022 was the year with the greatest amount of research.

**Table 2:** Distribution of publications by years.

Years	2010	2011	2012	2013	2014	2015	2016	2017	2018	2019	2020	2021	2022	2023	Total
**Number of articles**	11	16	22	28	36	56	62	55	45	80	91	86	110	49	750

### 3.2 Most productive sources

**Fig. 2:**
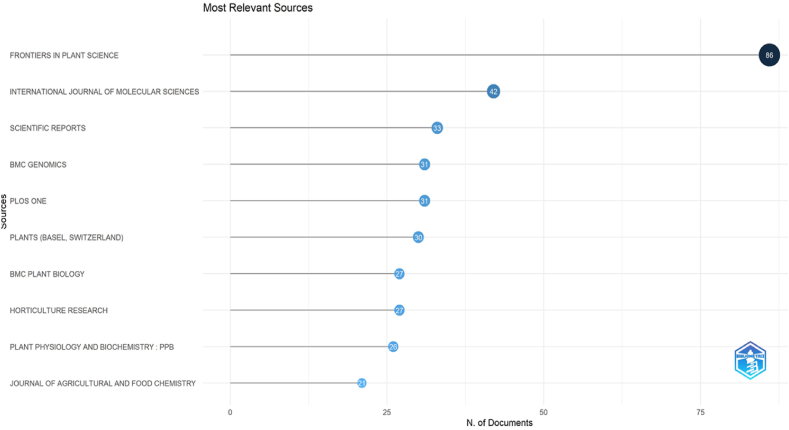
Number of documents most relevant sources.

With 86 articles in the top, Frontiers in Plant Science was the journal with the most publications on secondary metabolism in horticulture crops. This was followed by International Journal of Molecular Sciences, which had 42 articles. Scientific Reports, which had 33 articles. BMC Genomics and Plos One, which had 31 articles each. Plants (Basel, Switzerland) (f = 30), BMC Plant Biology (f = 27), Horticulture Research (f = 27), Plant Physiology and Biochemistry: PPB (f = 26) and Journal of Agricultural and Food Chemistry (21) ranked in the top 10 by publishing articles on the subject (Fig. 2).

### 3.3 Authors’ information

**Fig. 3:**
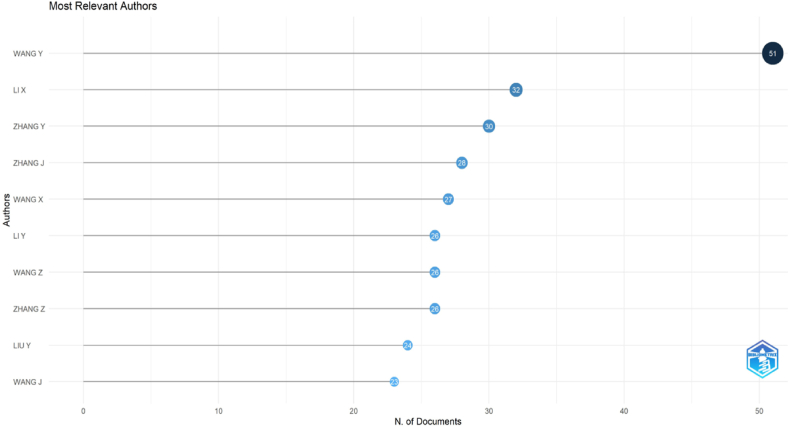
Most relevant authors.

When evaluated according to the number of publications made by the authors over time, the authors named Wang Y, Li X, Zhang Y, Zhang J, Wang X, Li Y, Wang Z, Zhang Z, Liu Y, and Wang J produced many publications, and information about this is given in Fig. 3. The authors that are rated in the top 10 have published 51, 32, 30, 28, 27, 26, 26, 24, and 23 times, respectively.

**Fig. 4:**
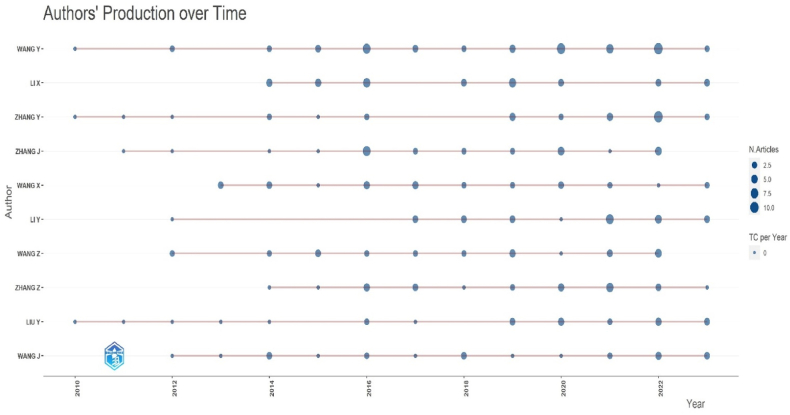
Authors' production over time.

When Fig. 3 are examined, the authors who published the most articles in SCI-Exp. indexed journals between 2010 and 2023 are listed as follows: Wang Y (51), Li X (32), Zhang Y (31), Zhang J (28), Wang X (27), Li Y (26), Wang Z (26), Zhang Z (26), Liu Y (24), and Wang J (23). These authors are the authors who most frequently contribute to a specific topic within “horticulture crop secondary metabolism” and are shown in Fig. 4.

**Fig. 5:**
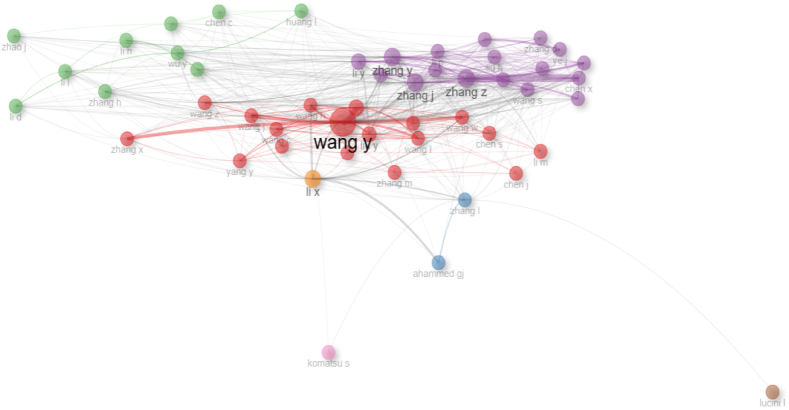
Collaboration network.

The collaboration network map between authors is shown in Fig. 5. Collaboration networks present publications made jointly by individuals or organizations, such as authors or journals, through a social network. The author x author adjacency matrix, which is essentially based on the frequency of co-publication, is employed in the collaboration network when author-based collaboration networks are analyzed. Seven distinct clusters were found to have developed when the network structure was studied. The writers were the nodes in the clusters, and the frequency of collaboration was indicated by the thickness of the links between the authors. The network's expansion of nodes indicates the writers' effect. When the clusters in the figure are examined, the author named Wang Y stands out in the red cluster. The authors collaborating with Wang Y were Wang X, Wang H, Wang Z, Liu Y, Wang J, Wang L, Wang H, Liu J, Chen S, Wang W, Yang Y, Li J, Wang C, Zhang M, Zhang X, Chen J, Li M, and Wang P. The author named Zhang Y in the purple cluster collaborated with Zhang J, Li Y, Zhang Z, Chen X, Li Z, Liu H, Zhang S, Chen Y, Zhang D, Zhang W, Liu W, Wang Q, and Ye J. In the green cluster, co-authors Wu Y, Huang I, Li H, Liu Z, Wang M, Li L, Zhang H, Chen C, Li D and Zhao J. Zhang L and Ahammed GJ were collaborated in blue cluster. Li X in the orange cluster, Lucini L in the brown cluster and Komatsu S in the pink cluster were separated from the other clusters by working individually.

**Fig. 6:**
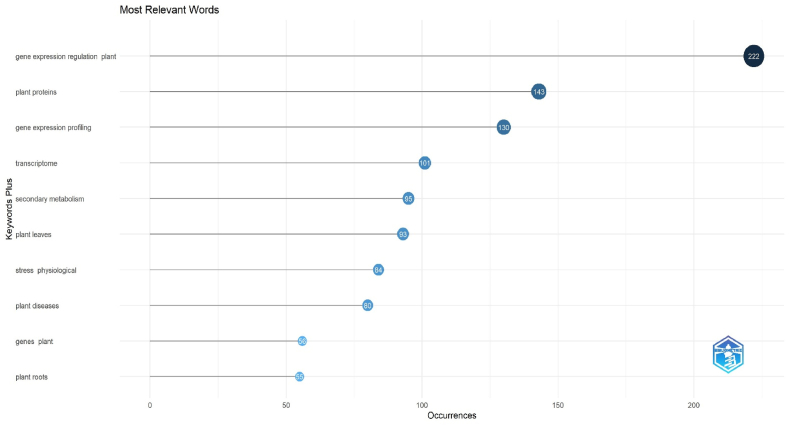
Most relevant words.

As a result of Web of Science-based scanning, SCI-Exp., the most frequently used keywords in the journals included in the index are presented in Fig. 6.

**Fig. 7:**
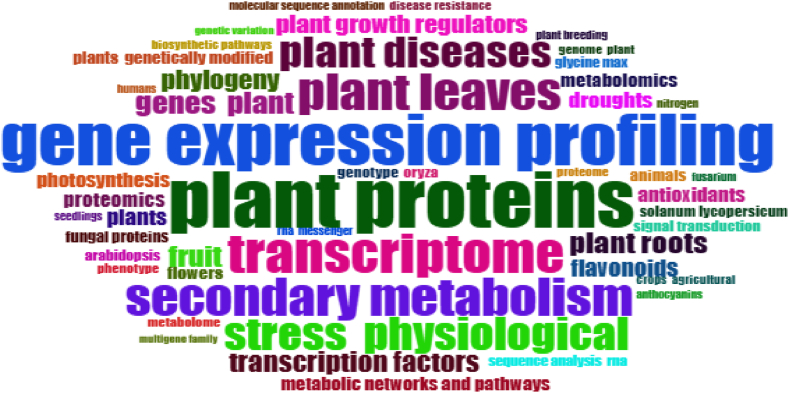
Word cloud.

As seen in Fig. 6, the most frequently used expressions in publications on the subject were "gene expression regulation plant", "plant proteins" and "gene expression profiling". These expressions were used 222, 143, and 130 times in the studies, respectively. These expressions were followed by the words “transcriptome (n = 101)”, “secondary metabolism (n = 95)”, “plant leaves (n = 93”), and “stress physiological (n = 84)”, respectively. The findings of a word cloud analysis conducted for the most popular terms are displayed in Fig. 7.

When Fig. 7 was inspection, it was revealed that the majority of the words were domain specific. The most used keyword was “gene expression regulation plant”. “Plant proteins” was the second most used keyword. The terms “gene expression profiling”, “transcriptome”, “secondary metabolism”, and “plant leaves” were mentioned after these.

### 3.4 Co-word network analysis

**Fig. 8:**
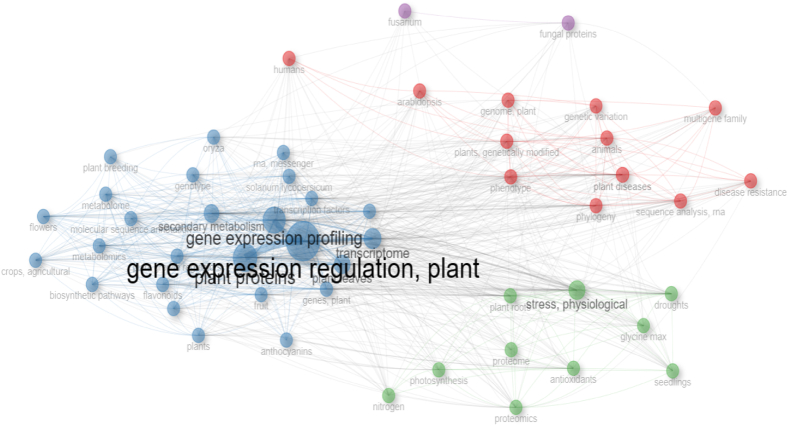
Collaboration occurrence network (keywords).

Fig. 8 displays the co-occurrence network among terms.

As seen in Fig. 8, a social network analysis of phrases related to each other was created. Subjects of the same color formed strong relationships with each other. The most strongly related topics were gene expression regulation, plant, transcriptome, gene expression profiling, plant proteins, stress, physiological, metabolic networks and pathways, solanum lycopersicum, genes and plant, flowers, gene expression regulation, genotype, plant growth regulators, and RNA, messenger. Stress, physiological, plant roots, droughts, proteomics, antioxidants, photosynthesis, glycine max, proteome, nitrogen and seedlings were closely related topics. Plant diseases, phylogeny, plants, genetically modified, animals, Arabidopsis, sequence analysis, RNA, phenotype, disease resistance, genome, plant, humans, genetic variation and multigene family were closely related topics. Fungal proteins and fusarium topics are also related to each other. Finally, a network map with a minimum of two links and 50 nodes that matched the requirements was created. Detailed information about the network analysis of the subjects studied is given in Supplementary Material 1.

### 3.5 Relevant affiliations and topics

When the institutions with which the authors of the published articles are affiliated were examined, the institution with the highest number of publications in China was the "Institute of Tropical Biosciences and Biotechnology, Chinese Academy of Tropical Agricultural Sciences (Catas), Haikou". There were 27 articles published by this institution. In second place was the "College of Horticulture and Gardening, Yangtze University, Jingzhou" institution in China, with 23 published articles. Information about this and other important institutions are presented in Table 3.

**Table 3:** Most relevant affiliations.

Affiliation	Articles
Institute of Tropical Biosciences and Biotechnology, Chinese Academy of Tropical Agricultural Sciences (Catas), Haikou 571101, China.	27
College Of Horticulture And Gardening, Yangtze University, Jingzhou, 434025, Hubei, China.	23
Key Laboratory Of Molecular Epigenetics Of Moe, Northeast Normal University, Changchun 130024, China.	17
State Key Laboratory Of Crop Stress Adaptation And Improvement, Plant Germplasm Resources And Genetic Laboratory, Kaifeng Key Laboratory Of Chrysanthemum Biology, School Of Life Sciences, Henan University, Jinming Road, Kaifeng, 475004, Henan, China.	17
Guangdong Key Laboratory Of Ornamental Plant Germplasm Innovation And Utilization, Environmental Horticulture Research Institute, Guangdong Academy Of Agricultural Sciences, Guangzhou, China.	16
Aquatic And Crop Resource Development, National Research Council Canada, Saskatoon, Saskatchewan, S7n 0w9, Canada.	15
Laboratoire Reproduction Et Dc) Veloppement Des Plantes, Univ Lyon, Ens De Lyon, Ucb Lyon 1, Cnrs, Inra, Lyon, France.	15
Institute Of Horticultural Biotechnology, Fujian Agriculture And Forestry University, Fuzhou, 350002, China.	14
Key Laboratory Of Horticultural Plant Biology (Ministry Of Education), College Of Horticulture And Forestry Sciences, Huazhong Agricultural University, Wuhan 430070, People's Republic Of China.	14
State Key Laboratory For Biology Of Plant Diseases And Insect Pests, Institute Of Plant Protection, Chinese Academy Of Agricultural Sciences, Beijing, China.	14

**Fig. 9:**
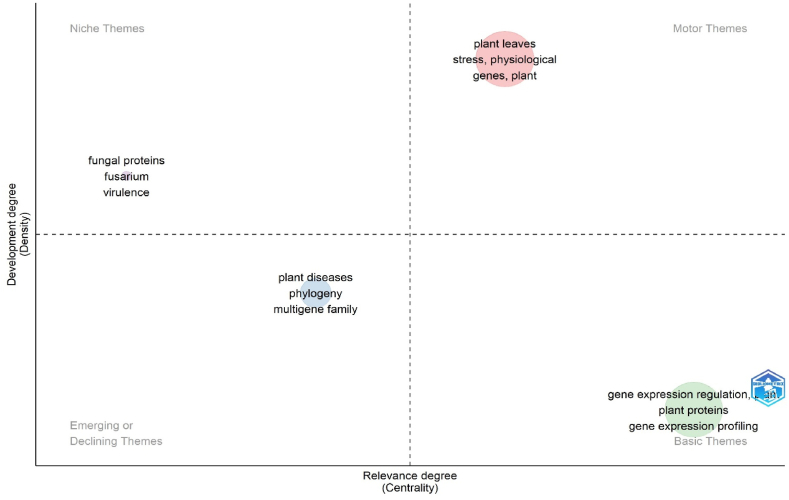
Thematic map.

Fig. 9 displays the topic densities representation. The most intensively studied topics were "gene expression regulation, plant", "plant leaves", "plant diseases", and “fungal proteins”. Supplementary Material 2 contains information about the pertinent thematic map clusters.

**Fig. 10:**
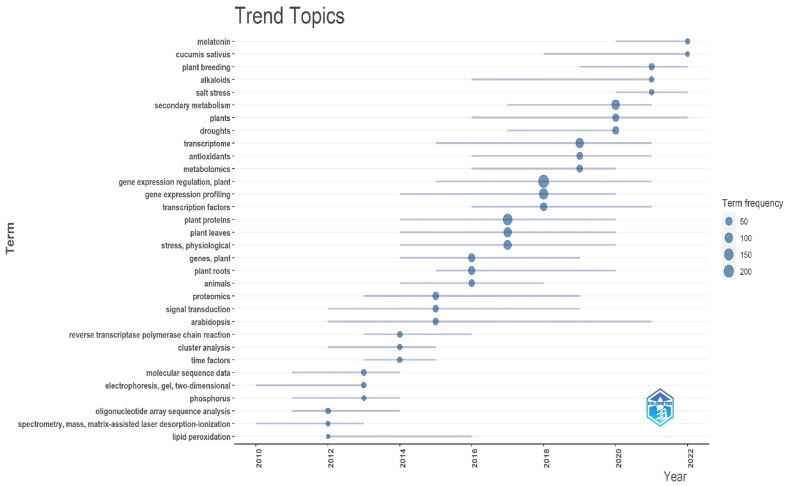
Trend topics of the field.

The ability to observe how keywords, titles, and abstracts have evolved over time is made possible by trend themes analysis, which has become a significant component in the growth of research in any field (Fig. 10). Terms on a coordinate plane are given logarithmic frequency values using trend topics analysis in order to track changes.

Fig. 10, which was created to show the words in the abstract, key word, title, and keywords mentioned at least three times each year between 2012 and 2022, was used to investigate the patterns of secondary metabolism research of horticultural crops. While research in recent years has regularly incorporated keywords from the gene expression regulation, plant, gene expression profiling, transcription factors, plant proteins, plant leaves, stress, physiological the phrases "arabidopsis", "melatonin", "cucumis sativus ", and "alkaloids" have retained their appeal.

### 3.6 Country/region networks

**Fig. 11:**
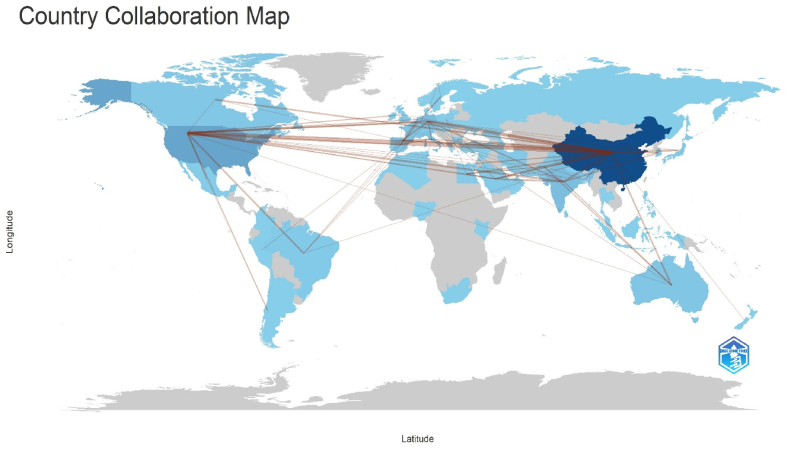
Collaboration world map.

The country/region networks display the concentration of paper production together with the relationships between the various countries and areas. Using the Bibliometrix program (version 4.3.2), the nation/region-based networks in the field of research on the secondary metabolism of horticultural crops are displayed in Fig. 11. The intensity of the blue color on the country map indicates the volume of publication activity, with darker blue indicating a higher number of publications. International collaboration is represented by the red connecting lines on the map; stronger lines indicate more frequent connections. Using the Bibliometrix tool (version 4.3.2), Table 4 presents the country/region collaborations in descending order of frequency of collaboration. With 47 collaborations, China and the United States had the largest level of collaboration, followed by China and Germany, and China and Pakistan, with 12 and 11 partnerships.

**Table 4:** The cooperation among countries.

From	To	Frequency
CHINA	USA	47
CHINA	GERMANY	12
CHINA	PAKISTAN	11
CHINA	INDIA	9
USA	INDIA	8
CHINA	FRANCE	7
USA	BRAZIL	7
CHINA	AUSTRALIA	6
CHINA	JAPAN	6
GERMANY	FRANCE	6

## 4 Discussion

Some research and their objectives can be summarized in light of the increase in the number of publications published between 2019 and 2022. Bulgari et al. [34] high-lighted advances in the recent development of biostimulant compounds, with a focus on their effects on enhancing tolerance to abiotic stresses in vegetable crops, in a study. Crops are frequently subjected to abiotic stressors throughout their lifecycle, which can have a significant impact on production and product quality. A calibrated blend of humic bioactive chemicals combined with microbial consortia may be a useful tactic to enhance crop productivity as well as nutritional and metabolic status, per the results of one study [35].

Horticulture Research was the journal with the most papers, as can be seen in Table 3 and Fig. 2. The studies published in this journal in recent years are briefly summarized as follows: Fan et al. [36] produced an article titled “A genome-wide association study uncovers a critical role of the RsPAP2 gene in red-skinned Raphanus sativus L.”, where they conducted a genome-wide association study of radish red skin color using whole-genome sequencing data generated from 179 radish genotypes. The R2R3-MYB transcription factor production of anthocyanin pigment 2 (PAP2) gene was discovered on chromosome 2 in a region associated with a leading SNP (single nucleotide polymorphisms). The amino acid sequence expressed by the RsPAP2 gene differed from that transcribed by the other RsMYB genes known to be important for the red skin col-or of radish. Overexpression of the RsPAP2 gene led to ATC accumulation in Arabidopsis and radish, as well as the activation of many ATC-related structural genes. A dual-luciferase reporter system and a yeast one-hybrid test revealed that RsPAP2 binds the RsUFGT and RsTT8 promoters. To investigate the association between theanine metabolism and N circumstances, Liu et al. [37] investigated the differentially ex-pressed genes (DEGs), proteins (DEPs), and microRNAs (DEMs) involved in theanine metabolism in tea plant shoots and roots. Tea plant shoots and roots were subjected to transcriptome, proteome, and microRNA studies under N sufficiency and deficit circumstances. Correlation studies were used to identify the DEP-DEG correlation pairings and the negative DEM-DEG interactions associated with theanine metabolism. These sequencing results were consistent with the expression profiles of DEGs and negative DEM-DEG pairings involved in theanine biosynthesis. Cluster analyses were conducted on the identified genes involved in theanine metabolism. The obtained results suggested that N adequacy and deficient situations had a substantial impact on the molecular and physiological mechanisms of theanine accumulation.

The second of the most important journals on the subject was Frontiers in Plant Science, and some of the studies published in this journal in recent years were as follows: MdJa2, a MADS-box transcription factor discovered by Su et al. [13], comprised a highly conserved MADS-box domain and belonged to the STMADS11 subfamily. The authors employed molecular and genetic approaches to investigate MdJa2's participation in the BR pathway and its regulatory effects on anthocyanin and PA production in red-fleshed apples. These findings give fresh light on the processes governing anthocyanin and PA accumulations in red-fleshed apples. Gui et al. [38] discovered that TSWV infected tobacco (Nicotiana benthamiana) plants in collaboration with HCRV, resulting in a higher infection rate for both viruses. They next employed the whole full-length transcriptome to investigate N. benthamiana's responses to TSWV-HCRV (TH) infection. They discovered that TSWV and HCRV infection elicited plant defensive responses such as the jasmonic acid signaling system, autophagy, and secondary metabolism. Overall, the findings shed light on the processes of TH co-infection in tobacco by using plant miRNA to lower plant basal resistance, and they led to the development of a novel technique for controlling crop disease caused by this viral complex.

As seen in Fig. 4 and Table 4, the results of the publications of the authors who published the most on the subject are summarized as follows: Wang et al. [39] published a gap-free nuclear genome and a complete mitochondrial genome of the bacterium U. virens JS60-2. A total of 222 effectors and 21 secondary metabolism gene clusters were predicted to aid in U. virens infection. The U. virens JS60-2 and its genomes will aid research into the U. virens rice pathosystem and aid in RFS regulation. CmWRKY53 was cloned from chrysanthemum and its expression was stimulated by aphid infestation in Zhang et al.'s [40] investigation. The findings revealed that CmWRKY53 mediates chrysanthemum aphid vulnerability. Secondary metabolite biosynthesis genes, such as peroxidase and polyphenol oxidase producing genes, were downregulated in CmWRKY53-overexpressing plants but substantially upregulated in CmWRKY53-SRDX plants. This revealed that the lower amounts of secondary metabolites in CmWRKY53 were related to chrysanthemum's sensitivity to aphids. Li et al. [41] identified the genes and miRNAs linked to the response to PVA infection in potato leaves using mRNA and small RNA sequencing. 201 miRNAs (DEMs) and 2062 differentially expressed genes (DEGs) were found to be present. These DEGs were implicated in hormone signaling induction, transcriptional reprogramming, pathogen signal transduction, activation of pathogenesis-related (PR) genes, and modifications to secondary metabolism, according to KEGG and Gene Ontology (GO) analyses. 58 miRNA–mRNA interactions related to PVA infection were discovered by small RNA sequencing. Certain miRNAs that target PR genes, such as stu-miR482d-3p and stu-miR397-5p, exhibited inverse associations with DEMs and DEGs. The results showed that disease-related genes were substantially expressed 60 h after PVA injection.

Interesting results have been drawn from the studies written on gene expression regulation, plant, transcriptome and gene expression profiling, which are among the most strongly interrelated topics shown in Fig. 8. ARD is a harmfully disrupted physiological and morphological reaction of apple plants to soils that have experienced changes in their (micro)biome as a result of prior apple cultures. Soil characteristics, faunal vectors, and trophic cascades influenced them, with genotype-specific effects on plant secondary metabolism, particularly phytoalexin production [42]. Jing et al. [43] investigated the impact of different light combinations on powdery mildew resistance and melon seedling development. Under blue light, H_2_O_2_ accumulated more, and the quantity of phenolics, flavonoids, and tannins, as well as the expression of genes involved in their production rose much more than in other treatments before and after infection. Melon treated with RB31 (3:1 red/blue light ratio) light had the best growth metrics. RB31 accumulated more lignin and had a lower incidence of powdery mildew than white light, red light, and RB71 (red/blue light ratio of 7:1). Blue light was found to boost melon resistance to powdery mildew, which is reliant on the stimulation of secondary metabolism, which may be connected to H_2_O_2_ buildup before infection.

In one study, the names of the top 10 journals related to cucumber research in the horticulture category and the top 10 journals in the same category in this study are completely different from each other. The results achieved in this study are different from each other due to the close collaboration of the authors with each other in the authorship network in the field of network visualization maps [44].

In another study [45], when Melon (*Cucumis melo* L) research in the horticulture category was compared with the top 10 journals in this study, it was found that only the "Journal of Agricultural and Food Chemistry" was in the top 10, and the other journals were different. The most productive authors, the institutions that publish the most articles, and the most frequently used keywords are completely different from the results in this study.

In Kolle et al.'s study [46], the institutions, journals and most productive authors that produce the most publications are different from the results reached in this study.

These differences may be due to scanning at different time intervals in other studies and the investigation of secondary metabolism in horticultural plants in this study. Because the second metabolism in horticultural has been an important issue that has attracted the attention of researchers in recent years.

## 5 Conclusion

As part of the study, bibliometric analysis of Web of Science database papers on secondary metabolism in horticulture plants was done. In the study, the data were obtained by searching for the phrase "horticulture crop secondary metabolism" in the Web of Science database on December 22, 2023. There were 750 publications in the Web of Science database covering the years 2010–2023. Of the 750 studies published in the Web of Science database; their distribution according to years, research areas, institutions, journals, and keywords was examined. The analysis discovered that academic works completed between 2010 and the present were grouped into a total of 4 clusters. The collaboration among authors in this topic is prevalent with an average of 7.64 authors per document. Furthermore, a word analysis of publications in the area revealed that the most often used words include gene expression regulation plant, plant proteins, gene expression profiling, transcriptome, secondary metabolism, plant leaves, stress physiological, plant diseases, genes plant and plant roots. This study also identified that the institution with the most research on Horticultural Crop Secondary Metabolism was in Institute of Tropical Biosciences and Biotechnology, Chinese Academy of Tropical Agricultural Sciences (Catas), Haikou, China. The cooperative structure of exceptional works, journals, and authors of horticulture plants secondary metabolism may be thought of as a roadmap that will serve as a starting point for future study.

## Funding

This research received no external funding.

## Data availability statement

The datasets created and analyzed during this study were obtained from the "http://127.0.0.1:7559/" website created with the biblioshiny(.) command in the RStudio program. Datasets are available from the corresponding author upon reasonable request.

## CRediT authorship contribution statement

**Şenol Çelik:** Writing – review & editing, Methodology, Investigation, Formal analysis, Data curation.

## Declaration of competing interest

Any no support provided to the author(s) or their institution(s) to cover salaries, consulting fees, and/or other expenses related to this study.
