# Supplementary material for: Bibliometric analysis of horticultural crop secondary metabolism
Source: Heliyon. 2024 Feb 14;10(4):e26079. doi: 10.1016/j.heliyon.2024.e26079 (PMC10881373; doi:10.1016/j.heliyon.2024.e26079)
Supplement: Multimedia component 1 [file mmc1.docx]

**Supplementary material**

**Supplementary Material 1.** Co-word network analysis

| **Node** | **Cluster** | **Betweenness** | **Closeness** | **Page Rank** |
| --- | --- | --- | --- | --- |
| Plant diseases | 1 | 19.37545686 | 0.016666667 | 0.027433158 |
| Phylogeny | 1 | 7.074040985 | 0.014705882 | 0.020505847 |
| Plants. genetically modified | 1 | 2.906910084 | 0.014705882 | 0.015073593 |
| Animals | 1 | 2.56195499 | 0.013888889 | 0.011781834 |
| Arabidopsis | 1 | 0.543243021 | 0.012987013 | 0.009761204 |
| Sequence analysis. RNA | 1 | 0.79352631 | 0.013888889 | 0.01321865 |
| Phenotype | 1 | 3.829662393 | 0.016129032 | 0.014351288 |
| Disease resistance | 1 | 0.665603146 | 0.012820513 | 0.01132766 |
| Genome. plant | 1 | 0.897385132 | 0.013157895 | 0.010283283 |
| Humans | 1 | 0.88057137 | 0.012658228 | 0.008136985 |
| Genetic variation | 1 | 0.601059336 | 0.012987013 | 0.009818222 |
| Multigene family | 1 | 0.397412341 | 0.012195122 | 0.008764348 |
| Gene expression regulation. plant | 2 | 152.0146138 | 0.02 | 0.09621831 |
| Plant proteins | 2 | 59.07686156 | 0.019230769 | 0.064842604 |
| Gene expression profiling | 2 | 63.42565098 | 0.020408163 | 0.061259347 |
| Transcriptome | 2 | 29.19633976 | 0.018518519 | 0.042446753 |
| Secondary metabolism | 2 | 39.35824502 | 0.02 | 0.035285749 |
| Plant leaves | 2 | 19.60063562 | 0.019230769 | 0.038271177 |
| Genes. plant | 2 | 8.949099107 | 0.017241379 | 0.028549135 |
| Fruit | 2 | 5.031190555 | 0.015384615 | 0.020902773 |
| Transcription factors | 2 | 7.808815715 | 0.016949153 | 0.025341794 |
| Plant growth regulators | 2 | 4.10676532 | 0.016129032 | 0.022557993 |
| Flavonoids | 2 | 5.834088332 | 0.016666667 | 0.021888858 |
| Plants | 2 | 1.052301183 | 0.01369863 | 0.011145726 |
| Metabolomics | 2 | 3.812580056 | 0.015873016 | 0.01576377 |
| Metabolic networks and pathways | 2 | 2.170676331 | 0.015384615 | 0.017091162 |
| Flowers | 2 | 0.709088427 | 0.01369863 | 0.01394954 |
| Genotype | 2 | 1.621577283 | 0.014084507 | 0.013978956 |
| Oryza | 2 | 1.311555675 | 0.013513514 | 0.013200675 |
| Signal transduction | 2 | 2.190524262 | 0.014285714 | 0.015210373 |
| Solanum lycopersicum | 2 | 1.568691316 | 0.014285714 | 0.013611025 |
| Metabolome | 2 | 0.973335186 | 0.014285714 | 0.012950566 |
| Biosynthetic pathways | 2 | 0.442939795 | 0.013513514 | 0.011510635 |
| Crops. agricultural | 2 | 1.762235083 | 0.014084507 | 0.011249225 |
| Anthocyanins | 2 | 0.008817866 | 0.011764706 | 0.008349092 |
| Molecular sequence annotation | 2 | 0.629007836 | 0.014084507 | 0.012119788 |
| Plant breeding | 2 | 0.154849858 | 0.012195122 | 0.007723249 |
| RNA. messenger | 2 | 0.973351218 | 0.014705882 | 0.014046116 |
| Stress. physiological | 3 | 21.29094357 | 0.017857143 | 0.040344759 |
| Plant roots | 3 | 8.495837185 | 0.016129032 | 0.024323849 |
| Droughts | 3 | 1.595452186 | 0.014492754 | 0.017809647 |
| Proteomics | 3 | 1.616247989 | 0.013888889 | 0.017708 |
| Antioxidants | 3 | 2.129947042 | 0.014285714 | 0.013717081 |
| Photosynthesis | 3 | 1.455064163 | 0.014285714 | 0.015919347 |
| Glycine max | 3 | 0.836796186 | 0.01369863 | 0.013446469 |
| Proteome | 3 | 1.189310756 | 0.012987013 | 0.010882323 |
| Nitrogen | 3 | 0.377342722 | 0.012658228 | 0.00931974 |
| Seedlings | 3 | 0.417675935 | 0.013157895 | 0.011895514 |
| Fungal proteins | 4 | 1.178944748 | 0.011764706 | 0.008321494 |
| Fusarium | 4 | 0.105774427 | 0.011235955 | 0.006391315 |

**Supplementary Material 2.** Thematic map clusters

| **Cluster** | **Callon Centrality** | **Callon Density** | **Rank Centrality** | **Rank Density** | **Cluster Frequency** |
| --- | --- | --- | --- | --- | --- |
| Plant leaves | 8.158303298 | 31.50230014 | 3 | 4 | 1725 |
| Plant diseases | 4.703327515 | 22.21552395 | 2 | 2 | 430 |
| Gene expression regulation, plant | 14.82977029 | 20.48817191 | 4 | 1 | 1674 |
| Fungal proteins | 1.550990659 | 23.08202864 | 1 | 3 | 144 |
